# Supplementary material for: Validation of Dietary Intake Estimated by Web-Based Dietary Assessment Methods and Usability Using Dietary Records or 24-h Dietary Recalls: A Scoping Review
Source: Nutrients. 2023 Apr 8;15(8):1816. doi: 10.3390/nu15081816 (PMC10141001; doi:10.3390/nu15081816)
Supplement: Supplementary file 1 [file nutrients-15-01816-s001.zip › nutrients-2287649 Supplemental.pdf]

Table S1. Reason for the excluded studies.

Review

	Exclusion reason	Reference number
1	Review, conference reports or abstract	[5,6,14,19-31]
2	Spanish (not written by English)	[51]
3	Not a validity study of nutritional intake	[52-58]
4	Not used the dietary records or 24-h dietary recalls for conventional or web-based dietary assessment methods	[59-68]
5	Not using face-to-face of traditional dietary methods	[69-77]
6	Not using internet of web-based dietary assessment methods	[78-89]
7	Not included the general population (pregnancy)	[90, 91]
8	Not assessed during whole one day	[92-97]

Hand search

	Exclusion reason	Reference number
1	Not a validity study of nutritional intake	[98]
2	Not used the dietary records or 24-h dietary recalls for conventional or web-based dietary methods	[99-101]
3	Not using internet of web-based dietary assessment methods	[102, 103]

Table S2. Score of including validity studies.

Variables		Score	Web-based dietary assessment methods: Dietary records							24-hour dietary recalls									
			Matsuzaki E et al. [35]	Monnerie B et al. [37]	Vereecken CA et al. [48]	Storey KE et al. [38]	Beasley J et al. [44]	Raatz SK et al. [46]	Teixeira V et al. [49]	Lafrenière J et al. [34]	Timon CM et al. [36]	Lindroos AK et al. [39]	Timon CM et al. [40]	Albar SA et al. [41]	Bradley J et al. [42]	Brassard D et al. [43]	Frankenfeld CL et al. [47]	Mescoloto SB et al. [50]	Liu B et al. [45]
1. Sample	Non-homogeneous sample (sex, SES, smoking and obesity)	0.5	0.5	0.5	0.5	0.5	0	0	0	0.5	0	0	0	0	0.5	0.5	0.5	0.5	0.5
	n . 100 (n . 50 for biomarkers)	0.5	0	0.5	0.5	0.5	0.5	0.5	0	0.5	0.5	0.5	0.5	0.5	0.5	0.5	0	0	0.5
2. Statistics																			
Group level	Compare/test mean or median or difference	1	1	1	1	1	1	1	1	1	1	1	1	1	0	1	1	1	1
Correlations	Crude correlation: 0.5 points Adjusted correlations (energy): 1.0 points Deattenuated or intraclass correlations: 1.5 points	0.5 - 1.5	1	0	0	1.5	0	0	0.5	1.5	1.5	1.5	0	1.5	0	0	0.5	0.5	0.5
Agreement	Classification or Bland & Altman plot	0.5	0.5	0	0	0	0.5	0.5	0.5	0.5	0.5	0.5	0.5	0.5	0.5	0	0.5	0	0.5
3. Data collection	Gathered by face to face interview	1	0 [a]	1	0 [b]	1	1	1	1	1	1	1	1	1	1	1	0 [b]	1	1
4. Seasonality	Considered	0.5	0	0	0	0	0	0	0	0	0	0	0	0	0	0	0	0	0
5. Supplemetns	Included and data considered in analysis	1.5	0	0	0	0	0	0	0	0	0	0	1.5	0	0	1.5	0	0	0
	Total	Min:0 Max:7	3.0	3.0	2.0	4.5	3.0	3.0	3.0	5.0	4.5	4.5	4.5	4.5	2.5	4.5	2.5	3.0	4.0

[a] The conventional dietary record was non-face-to-face.

[b] The conventional dietary record did not describe face-to-face.
